# Productivity Effect Evaluation on Market-Type Environmental Regulation: A Case Study of SO_2_ Emission Trading Pilot in China

**DOI:** 10.3390/ijerph17218027

**Published:** 2020-10-31

**Authors:** Yanhong Feng, Shuanglian Chen, Pierre Failler

**Affiliations:** 1 School of Economics and Statistics, Guangzhou University, Guangzhou 510006, China; 1111864008@e.gzhu.edu.cn; 2 Guangzhou International Institute of Finance and Guangzhou University, Guangzhou 510006, China; 3 Economics and Finance Group, Portsmouth Business School, University of Portsmouth, Portsmouth PO1 3DE, UK; pierre.failler@port.ac.uk

**Keywords:** environmental regulation, emission trading, TFP, PSM-DID

## Abstract

Taking China’s SO_2_ emissions trading pilot (ETP) in 2007, a large-scale market-based environmental regulation as its target, this paper reexamines the strong Porter hypothesis by adopting the method of propensity score matching-differences-in-differences. Research shows the following results: first, SO_2_ ETP which provides high flexibility for enterprises in the process of emission reduction, improves total factor productivity (TFP) significantly on the whole. Second, the productivity effect of market-based environmental regulation varies from the productivity level of enterprise. For example, the SO_2_ ETP has a significant effect on TFP only at 40–80 percent quantile of TFP, and the effect increases at first and then decreases. Third, the financing constraints and bargaining power of enterprises have significant negative moderating effects on the impact of SO_2_ ETP on TFP, and the moderating effects between state-owned and non-state-owned enterprises exist heterogeneity. In conclusion, it provides reference for the formulation of market-type environmental regulations and the realization of high-quality development for developing countries.

## 1. Introduction

With the acceleration of economic growth and industrialization, the increasing contradiction between energy supply and demand, as well as the overdraft of environmental bearing abilities, have brought severe challenges to global environmental protection and sustainable development [1,2]. Especially, the increasing SO_2_ emissions have become the main source of air pollution [3]. As the world’s largest SO_2_ emissions country for a long time [4], the Chinese government has carried out a series of environmental policies to control SO_2_ pollution emissions. Recently, China has begun to advocate to achieve pollutant emission reduction and environmental improvement by making use of market effectiveness. For example, they have promoted the trading system of carbon emission rights, water rights, and energy use rights vigorously in order to develop environmental protection market. Emission trading system is a system in which the resource value is given to environmental quantity, the emission right is determined and permitted to transfer. Compared with the previous command and control environmental regulation, these systems are considered to provide enterprises with more flexible mechanisms to protect the environment. Meanwhile, it even increased the productivity through production processes adjustment, reallocation of resources and innovation [5]. For SO_2_ emissions reductions, America is the first one to implement the world SO_2_ emissions trading pilot (ETP) since 1990. China has begun to implement large-scale SO_2_ ETP in 2007 by learning from the successful experience of international market-based environmental regulation. Simultaneously, 11 pilot provinces and cities were approved, including Jiangsu, Tianjin, Zhejiang, Hubei, Chongqing, Hunan, Inner Mongolia, Hebei, Shaanxi, Henan, and Shanxi. In 31 provinces and cities of national total, the number of pilot provinces accounts for nearly one-third. Since the implementation of SO_2_ ETP, much attention has been paid to its environmental effects, and there is a consensus that SO_2_ ETP has an important significance with SO_2_ emissions reducing, cleaner production promoting, as well as environmental protection.

However, it is difficult to maintain the coherence between environment and economic. Does this kind of market-oriented environmental regulation have economic effect in the process of implementation in developing countries? Is that beneficial to the improvement of enterprise productivity? Currently, there still exist some controversies on these questions. Typically, Martin et al. [6] explored whether the European Union (EU) Emissions Trading Scheme (ETS), the first and largest carbon market in the world, has an impact on the economy. They concluded that there is a clear negative impact on emissions reductions, but no evidence supports the view that the EU ETS has strong detrimental impacts on economic performance. Focusing on the Germany data, Oestreich and Tsiakas [7] studied the effects of EU ETS on stock return and found that enterprises with carbon emission allowances have higher profits than those without. Tang et al. [8] took China’s SO_2_ ETP in 2007 as a natural experiment to carry on the empirical research by adopting the data of Chinese manufacturing companies. The results showed that Chinese SO_2_ ETP promoted enterprise innovation significantly, but without any effect on enterprise total factor productivity (TFP). While, Peng et al. [5] found that market-based environmental regulation has a significant productivity enhancement effect for all types of industrial enterprises, and the effect is more obvious for private enterprises, and more productive and less polluting enterprises. In fact, the view of Peng et al. [5] is also supported by other scholars. For example, Porter hypothesis held that environmental regulations may hinder productivity growth [9,10], while, well-designed regulation triggers innovation partially or completely, offs ETP compliance costs, and improves the TFP; Schleich and Betz [11] agreed that the emission trading system has a positive impact on the emission reduction for small and medium-sized enterprises, by employing the method of experimental simulation; Testa et al. [12] confirmed that the significant increasing proportion of R&D investment under the more flexible environmental regulation in the EU construction industry, improves the production efficiency of enterprises. Therefore, under the dual background of environmental protection and high development, re-examining the impact of market-based environmental regulation on TFP by taking China’s largest SO_2_ ETP practice as an example is a very important practical problem. On the one hand, the discussion of this issue could support China’s recent transition from traditional environmental regulation to market-based means. On the other hand, it provides reference for the formulation of market-based environmental regulation and the realization of high-quality development in other developing countries.

This paper takes China’s largest market-type environmental regulation—SO_2_ ETP in 2007 as a quasi-natural experiment, and makes research on the impact of SO_2_ ETP on TFP by the method of propensity score matching-differences-in-differences (PSM-DID). On this basis, it used the unconditional panel quantile fixed effect regression to test the heterogeneity effects of SO_2_ ETP on enterprises TFP at different levels of TFP. Then, we further investigate the moderating effects of corporate financing constraints and bargaining power on the relationship between SO_2_ ETP and enterprise TFP.

The study may make four contributions to the existing literature as follows: First, taking the market-type environmental regulation as its research object, this paper re-examines the applicability of the strong Porter hypothesis from the aspects of micro-enterprise. First, Porter assumes that environmental regulations stimulate innovation and enhance competitiveness, as well as enhance enterprise TFP [10], which has been confirmed by many researchers [13,14,15,16,17]. However, some other scholars also think that environmental policy, as a regulatory pressure, means extra costs and have a negative impact on the TFP [18,19,20]. Furthermore, there are other literatures showing that the impact of environmental regulation on enterprise TFP is unclear [8,21,22]. So, Porter hypothesis still remains controversial. Then, most previous studies did not distinguish imperative or market-based environmental regulation tools. The intensity of environmental regulation is usually defined by environmental control expenditure, government environmental subsidies, pollutant emission standards [23,24,25,26,27], and environmental policy index [15,28], or replaced by regulatory target [29,30]. However, there are few studies or no agreement conclusions on the market-based environmental regulation [8,31]. Furthermore, from the literature available, related studies on ETP mainly focused on developed countries such as the United States and Europe [11,32,33,34], but ignored the developing countries. In addition, most related studies concentrate on the macroeconomic level that ignores the heterogeneity of the enterprise, which may make the results appear biased [35,36]. Therefore, this paper takes SO_2_ emission enterprises in China’s listed manufacturing enterprises as the object, and finds out that the practice of SO_2_ ETP in China shows a strong Porter effect on the whole. This result not only supports the applicability of the Porter hypothesis, but also provides supplement for the existing literature.

Second, this paper testes the heterogeneity of market-based ETP on enterprise TFP at different levels of TFP by using unconditional panel quantile fixed effect regression. In face with ETP policy, the business activities of enterprises at different TFP levels, such as production, investment, and innovation, show obvious differences, which lead the different degree effect of ETP on TFP. However, this heterogeneous effect is rarely studied and still remains controversial. Typically, Albrizio et al. [15] concluded that stricter environmental regulation will increase the productivity by 20% for high-productivity enterprises, but decline the productivity for low-productivity enterprises by grouping samples according to different TFP levels. In contrast, Peng et al. [31] pointed out that the preliminary exploration of SO_2_ ETP in 2002 has positive contribution to the TFP of different quantile enterprises. To be specific, the positive effect for lower TFP productivity is relatively weak, but it significantly strengthens for higher TFP companies. Thus, based on unconditional panel quantile fixed effect regression method from Firpo et al. [37] and Borgen [38], this paper continues to test the heterogeneity effect of SO_2_ ETP on TFP. This method can show the marginal influence of explanatory variables on unconditional quantile of explained variables, under the premise of ensuring accurate identification of model and unanimous parameters estimation [39,40]. Therefore, it makes DID model keep in line with the general meaning of policy assessment.

Third, this paper examines the moderating effects of corporate financing constraints and bargaining power on the relationship between SO_2_ ETP and TFP, respectively. On the one hand, enterprises tend to have motivation and ability to carry out technological innovation-related activities when their financing constraints are in good condition [41,42]. Then further, technological innovation improves quality and efficiency through knowledge spillover effect. In other words, the financing constraint has a moderating role on the relationship between ETP and enterprise TFP. On the other hand, for polluting enterprises, they are likely to make greater contribution to the local economy and officials’ performance assessment when they get higher output value, pay greater total tax payment, and provide more jobs. Based on that, they obtain higher bargaining power of the government in the process of environmental regulation implementation [43,44]. Hence, it may reduce the TFP effect of environmental regulation. However, the existing studies do not pay particular attention to the impact of these factors on the effectiveness of TFP policy implementation. Therefore, through empirical research, this paper finds that the financing constraints and bargaining power of enterprises have a negative moderating effect on the impact of SO_2_ ETP on TFP. It could provide reference and guidance for market-type environmental protection policies to better improve TFP.

Fourth, this paper improves its empirical method and sample selection when studying the impact of ETP on enterprise TFP. At first, this paper takes China’s largest SO_2_ ETP in 2007 as a natural experiment and uses PSM-DID method to carry on the empirical study on the exogenous shock. It not only reduces the effect of grouping non-randomness, estimation bias, and confounding variables, but also avoids endogenous problems, which makes the estimates more rigorous [45,46]. Second, a new method is used to measure the enterprises TFP. Differently from usual, we adopt an improved dynamic panel instrumental variables method to measure the enterprise TFP. The method is proposed by Rovigatti and Mollisi [47]. Referencing to Blundell and Bond [48], they added the lag term on the basis of generalized method of moments (GMM) estimation from Wooldridge’s framework [49]. This improvement can increase moment limits without losing information, then get more robust results. In addition, the SO_2_ emission enterprises in Chinese listed manufacturing enterprises are selected for empirical sample. Previous micro-level researches are generally based on all heavily polluting enterprises or manufacturing industries. However, they ignored the policy objectives of SO_2_ ETP and the limitations of the measurement method of enterprise TFP. Actually, SO_2_ ETP is aimed at SO_2_ emission reduction, which mainly affects the environmental and business behavior of SO_2_ emission enterprises. Moreover, studies have shown that existing methods of TFP measurement are not applicable to mining and oil companies and other industry such as production supply of hydropower and gas [50,51].

The remainder of this paper is organized as follows. Section 2 is the policy background and research hypothesis. Section 3 is the empirical strategy, including model setting, sample, and data. Section 4 is the empirical results, including the basic regression results and robustness test. Section 5 is heterogeneity analysis and moderating effect analysis. The conclusions and policy recommendations are shown in Section 6.

## 2. Policy Background and Research Hypothesis

### 2.1. Policy Background

Under the extensive development model, high SO_2_ emission becomes the main source of air pollution. In the 1990s, the Chinese government began to pay more attention to regional air pollution control in response to environment deteriorating. They implemented some environmental policies, such as: The Law of Prevention and Control of Air Pollution in People’s Republic of China, which was promulgated in 1987 and then amended for SO_2_ emission problem in 1995 and in 2000 respectively. At the same time, the State Council approved and implemented the policy of Division Program of Acid Rain and SO_2_ Two Control Zone (TCZ) in 1998. In 2002, the Ministry of Environmental Protection issued a technical policy on prevention and control of SO_2_ emissions from coal-fired power plants, large-scale industrial boilers and kilns, and urban civil stoves. In 2004, the implementation of Pollutant Emission Standards for Thermal Power Plant Air set more stringent standards for SO_2_ emission. However, the traditional way that governments rely on command and control rules heavily, may be effective but often less efficient. On the one hand, the implementation cost is huge since the regulation is directly implemented by the government, and the effect depends on the degree of policy implementation and enterprises’ acceptance. On the other hand, the one-size-fits-all way that forces enterprises to achieve environmental compliance, just limits the enterprises’ enthusiasm and pollution controlling flexibility.

In the early 20th century, the cooperative program of Study on Promoting Total Emission Control of SO_2_ Emissions and ETP Implementation between China and America has begun the initial exploration of emission permit trading system in China. Then in 2002, in order to carry out the research on compensated use and trading system of SO_2_ emission rights, the Ministry of Environmental Protection launched a “4 + 3 + 1” project for this task. The project includes Shandong, Shanxi, Jiangsu, and Henan provinces, and the cities of Shanghai, Tianjin and Liuzhou, and Huaneng Group. To some extent, it points out the direction for getting rid of the dilemma of pollution first and treatment afterwards. Although these trading pilot areas are typical, the ETP preliminary exploration is a great limitation. For the one part, their scope is mainly limited to the power industry in some cities; for the other part, these pilot areas have neither set up emissions-trading center, nor formed the emissions-trading market.

After the preliminary exploration in 2002, Central Government officially launched a large-scale trading polit of SO_2_ emission rights in 2007. The number of pilot provinces is 11, which includes Jiangsu, Tianjin, Zhejiang, Hebei, Shanxi, Chongqing, Hubei, Shaanxi, Mongolia, Hunan, and Henan. In general, the China’s SO_2_ ETP is jointly implemented by the central government and local government and market mechanism, and its main rules are as follows: first, the pilot scope is uniformly designated by the Environmental Protection Ministry. These 11 designated regions are uniformly distributed in the eastern, central, and western regions, which represent different levels of economic development. Among them, the eastern, central, and western regions have 4, 4, 3 provinces respectively. From the pilot scale, in 31 provinces of national, the number of pilot provinces accounted for 35.5%; in 2007, the GDP and industrial SO_2_ emissions of pilot areas accounted for 42.8%, 50.2% of the national total, respectively. Second, the emission rights are distributed from the central government to local governments. Generally speaking, first, the Environmental Protection Department prescribes the total amount of emissions (the upper limit) in a certain period (usually five years). Then, they allocate emissions to each province based on the actual emissions of each province every year (or the “permit”). The total number of quotas issued is equal to the total emission upper limit. Meanwhile, the quota distribution method is also applied in cities in each province and enterprises in each city. Moreover, the price of SO_2_ emissions trading is led by the market, and assisted by the government. The governments set benchmark prices for SO_2_ emissions trading; but its actual transaction price is mainly regulated by the market. Emission enterprises can purchase or sell quotas at regional emission trading centers according to actual needs. For the place that has not established trading center, the pollution emission enterprises may also trade through other means prescribed by the government. A common mean is direct trading among emission units and government environmental management departments and emission enterprises.

Because of current market mechanism, developed internet and governments’ efforts, these pilot areas have set up emission trading centers and established standard operating procedures successively. In 2014, general office of the State Council issued Guiding Opinions on Further Promoting the Work of Compensated Use of Emission Rights and Trading Pilot. Then, SO_2_ ETP were further promoted nationwide in 2017. Overall, the implementation of SO_2_ ETP in 2007 has achieved some remarkable results. Up to 2012, almost all the pilot areas have established provincial or municipal emissions trading centers, as well as their emissions trading management measures. Actually, the pilot transactions have reached more than 4 billion RMB up to 2013. According to the data of China Environment Statistics Yearbook, SO_2_ emissions of every province pilot has decreased obviously [8].

### 2.2. Research Hypothesis

The Porter hypothesis and its proponents argue that strict and flexible environmental regulation stimulates firms to innovate their technology and promotes TFP growth [20,52,53]. ETP, as an important market-based regulatory tool, is characterized by its clear market price signal and pollutants focus. As we know, the emission right has the property right attribute and the profit of enterprise is equal to the income (including the sale of emission rights and green innovation, etc.) minus the cost (including purchasing emission rights and green innovation, etc.). So, driven by the biggest profits, the companies make decisions about whether to invest innovation or buy permits based on their circumstances. In general, ETP affects TFP mainly in two ways. The first way is that ETP policy promotes the polluting enterprises’ technological innovating and upgrading [54], and then improves the overall TFP. To be specific, first, as an environmental legitimacy regulation, ETP makes enterprises increase the expected cost of pollution control and production. Hence, on the whole, in pursuit of profit maximization, the firms choose to improve technology and reduce production costs [55]. Second, under the implementation of ETP, the qualified enterprises would not choose to discharge pollutants under the quota at random, but to store the surplus emission quota in a planned way for future use or to be sold for a profit. Thus, the economic gains from the sale of emission permits can provide a growing economic incentive for enterprises to undertake improvements in pollution control and clean production technologies [56]. Third, based on the market mechanism, ETP provides enterprises with more market information for technological innovation and improvement [57], which reduces the risks and uncertainties in technological innovation. As we know, the promotion of the technological innovation and upgrade means the improvement of the overall TFP [58,59].

In addition, ETP influences the overall TFP through improving the resource allocation efficiency. On the one hand, by providing greater flexibility in the process of emission reduction [15], ETP policy optimizes the allocation of emission quantity among enterprises with different emission reduction costs. On the other hand, as a kind of environmental regulation, ETP bring certain pressure cost for enterprises. Thus, enterprises reconsider the allocation of production factors. For instance, they would reduce the input of polluting and inefficient production sectors, and increase the investment in clean and efficient production sectors for the sake of long-term economic benefits [50,51]. Based on that, it is reasonable to hypothesize that:

**Hypothesis** **1.** *SO_2_ ETP improves the overall TFP significantly*.

Generally speaking, the different TFP level usually means the different technical level, production efficiency, and innovation ability of enterprises themselves. Thus, the operation activities of enterprises with different levels of TFP, such as production, investment, environmental protection expenditure, and innovation behavior, are quite different when facing up to ETP. Then, for enterprises with different TFP levels, ETP may have heterogeneous effect on their TFP. In general, when the enterprise TFP level is low, its overall technical level is also not optimistic. At this moment, the enterprises may make two decisions in response to ETP. On the one hand, if the enterprises have confidence in improving the technological innovation, they choose to strengthen the technological innovation. But they may cannot realize their economic effects because technological innovation requires a large amount of costs, which cannot be offset by the benefits. On the other hand, if the enterprises have no confidence in that innovation, they will choose to bear the costs of environmental governance but without motivation to invest more on innovation. Therefore, the impact of ETP on TFP for low TFP firms is weak or not obvious [15]. With the increase of TFP, the pulling effect of ETP on enterprise TFP is strengthened. For the enterprises with a certain TFP level, their environmental regulation cost may be offset by the gains in productivity. Hence, facing up to TFP, the enterprises will choose to increase innovation input and improve the level of technological innovation in order to reduce the cost of emission reduction or gain emission benefits. Then, TFP can be improved. However, when TFP rises to a certain degree, the green technical innovation ability of enterprises becomes very strong, and their emission technology gets advanced, but it has difficulty to make a big breakthrough in the technology. At this time, enterprises have sufficient capacity to deal with pollution emissions, but the input costs of further improving clean production and pollution treatment technology is more than the proceeds from emission rights sale. As a result, the enterprises no longer choose to carry out green technology innovation activities. Then, the impact of TFP on their ETP becomes less significant. Therefore, we formulate the following hypothesis:

**Hypothesis** **2.** *SO_2_ ETP have heterogeneous effects on TFP at different levels of TFP*.

Financing constraint can be understood as difficult degree of obtaining funds and the cost of financing. It is an important factor that influences the environmental strategic behavior of an enterprise, and may also affect practical activity of enterprises by distorting the optimal allocation of production inputs [60]. Thus, the productivity effect of government environmental protection policy will be affected by enterprises’ own financing constraints. In general, the moderating mechanism of financing constraints is related to enterprise technological innovation. On the one hand, because of the high opportunity cost and high initial input with low return on investment of technological innovation, the R&D activities of enterprises are more likely to get into financing difficulties than other activities [61]. Therefore, when facing the fact that ETP makes higher cost of pollution control, enterprises with different financing constraints react differently. The enterprises with serious financing constraints would give priority to productive investment. In such circumstance, in order to obey the regulation, they only increase expenditure to achieve the end treatment of pollution or purchase emission rights, but do not have motivation to engage in relevant R&D activities [62]. On the contrary, the enterprises with less financing constraints may choose to have stronger social responsibility, consciousness of environmental protection and innovation. Thus, it makes their expectation on innovation effect of environmental regulation become clearer. Moreover, with the sufficient financial support, the enterprises are more determined to meet the challenge of environmental regulation and promote the enhancement of their core competitiveness through technological innovation. On the other hand, the implementation of environmental policy is often combined with the credit system. Through this way, the enterprises who fulfill their social responsibilities, gain credit support from banks easily. Then, they can ease the financing constraints and promote technological innovation activities. Because of the strong knowledge spillover effect of technological innovation, it can improve the efficiency of resource utilization and thus increase TFP. Based on that, we propose another hypothesis:

**Hypothesis** **3.** *The positive impact of SO_2_ ETP on TFP gets smaller when the financial constraints of the enterprises become higher*.

Based on current supervision mechanism, Chinese government has certain power to decide the appointment and removal, and funds allocation in the local environmental protection departments. Moreover, they also own the ability to influence the enforcement of environmental regulations because of information asymmetry. Thus, the government gives pollute enterprises a chance to bargain [63]. In addition, economic performance, as a core criterion for current promotion system, may provide incentives to weaken environmental policy implementation for local governments [43]. Actually, a large number of officials choose to give priority to developing economic and solving employment, and make compromises in exchange for short-term economic growth when the economy and the environment cannot be balanced. Even, in order to complete economic performance appraisal, as well as stabilize local government revenue and ensure the normal operation of public services, the local governments try to help enterprises within its jurisdiction avoid the impact of environmental regulation policies on their production, operation, and relocation. As a result, for the heavily polluting enterprises, if they make large contribution to local economic growth and employment, they gain more privileges on negotiating special right with local governments even when facing up to strict environmental regulation policies [44]. For example, in the implementation of ETP, enterprises with high bargaining power tend to be allocated more emissions or little pollution monitoring or got some implicit tax subsidies, which makes the enforcement of ETP become weak or unfair. So, the heavy polluters with high bargaining power can be exempted from environmental regulations to a greater extent, and save some cost of pollution discharge. Further, these enterprises have no motivation to improve their technological innovation level, which leads to weakening of the productivity effect of environmental regulation. Therefore, we hypothesize that:

**Hypothesis** **4.** *The positive impact of SO_2_ ETP on enterprises TFP is smaller when the bargaining power of the firm is higher*.

## 3. Empirical Strategy

### 3.1. Empirical Model

The China’s SO_2_ ETP in 2007 provided a quasi-natural experimental environment for the economic effects of market-based environmental regulation. For effect evaluation of the policy, DID method is one of usual method. By comparing the difference between pilot provinces (treated group) and non-pilot provinces (control group) for SO_2_ emission right trading in 2007, a benchmark DID model is constructed:

(1) $$TFP_{it}=\beta_{0}+\beta_{1}time_{t}*treated_{i}+X_{it}\lambda +\mu_{i}+\theta_{j}+\upsilon_{k}+\gamma_{t}+\varepsilon_{ijkt,}$$

where subscripts i, j, k, and t represent the enterprise, industry, area, and year, respectively. $TFP_{it}$ means the TFP of enterprises i in period t; $\beta_{1}$ is the net effect of policy implementation; variables of time and treated mean whether the sample is in the policy implementation period and belongs to the pilot area. They are expressed by two dummy variables. In 2007, the central government officially launched policy on the compensated use and trading pilot of SO_2_ emission rights, and has approved 11 SO_2_ emission trading pilot provinces, such as Jiangsu, Tianjin, Zhejiang, Hebei, Shanxi, Chongqing, Hubei, Shaanxi, Inner Mongolia, Hunan, Henan. According to that, we let time=0 if the time of sample is before 2008, otherwise, let time=1. Meanwhile, if the sample enterprises belong to the 11 pilot areas, let treated=1, otherwise, let treated=0 (20 provinces in total). X represents a range of control variables. λ is a regression coefficient matrix. In addition, $\mu_{i}$, $\theta_{j}$, $\upsilon_{k}$, and $\gamma_{t}$ are individual fixed effect, industry fixed effect, area fixed effect, and time fixed effect, respectively. $\varepsilon_{ijkt}$ is a random error, which satisfies the assumption of $E(\varepsilon_{ijkt}|time*treated,X_{it},\mu_{i},\theta_{j},\nu_{k},\gamma_{t})=0$.

In order to ensure sufficient comparability between the treated and control group, following the ways of Abadie et al. [64] and Lu [65], this paper uses propensity score matching (PSM) before adopting the DID method. Because the initial conditions of these two groups may cause selection bias, it needs to use PSM method to re-group and eliminate the sample that does not meet the requirements. This method can reduce the influence of the unsatisfied randomicity of the experimental group matching, estimation bias, and confounding variables, and then avoid an endogenous problem. Specifically, this paper uses the Logit regression to estimate the propensity score, and uses the nearest-neighbor matching method to match.

### 3.2. Sample and Data

The sample is based on the data of the SO_2_ emission enterprises in Chinese listed manufacturing enterprises from 2004 to 2015. The selection process is as follows. First, it has three steps on the enterprises’ selection. The first step is to target the sample within the manufacturing sector, which is considered from two facts. For the one fact, the SO_2_ emission are mainly concentrated in steel, cement, and other manufacturing industries, mining and electricity industries as well. For the other fact, the emission permits of electricity industry are traded only within their own sector and the analysis methods of existing TFP are not applicable to mining, oil companies, and other sectors of water and electricity gas production and supply [50,51]. Based on this, the second step is to determine the SO_2_ emission enterprises according to the information from annual corporate report and Corporate Social Responsibility Report of the listed companies. Specially, the enterprise is identified as SO_2_ emission enterprises as long as it contains any relevant information of the established criteria. In the third step, in order to guarantee the stability and validity of the samples, it needs to weed out the enterprises of suffering from continuous losses (called ST and * ST) and with serious missing value. Through the screening, 260 SO_2_ emission enterprises involving 33 industries are finally obtained, with 101 in pilot provinces and 159 in non-pilot provinces. Second, for the selection of sample period, since the SO_2_ ETP started in 2007, and was fully implemented in early 2017, this paper selects the data from 2004 to 2015. In addition, in order to eliminate the interference of other factors and to test the robustness of the evaluation results, a differences-in-differences-in-differences (DDD) model is constructed. All the listed companies in non-SO_2_ emission industry are used as a sample for another treated and control group. A total of 257 enterprises are obtained after weeping out the ST and * ST Enterprises and any others with serious missing value. In the aspect of data sources, the enterprise data used in this paper are all from CSMAR database, and the provincial data in the control variables are from the China Statistical Yearbook.

### 3.3. Measurement of Main Variable TFP

Different from the usual semi-parametric methods of Olley-Pakes (OP) and Levinsohn-Petrin (LP) [66,67], the new improved method provided by Rovigatti and Mollisi [47] could increase the moment limit without loss of information, thus making the results more robust.

First, considering the Cobb-Douglas production function:

(2) $$y_{it}=\alpha +w_{it}\beta +x_{it}\gamma +\omega_{it}+\varepsilon_{it}$$

where y is the logarithm of the added value, which is measured by industrial added value from income calculation (donated in hundred Yuan), as well as reduced by the ex-factory products price index from industrial added value. w is the logarithm of labor force number (donated in thousand), which is calculated by the logarithm of enterprise worker number. x is the logarithm of capital (donated in hundred Yuan), which is measured by net fixed capital and deflated by its price index. ω is the total factor productivity (TFP) that is unobservable. ε is a white noise.

The enterprises intermediate input m is measured by income method. Under the perfect competition where input and output prices are identical across firms, we write the functional expression of intermediate input demand function as

(3) $$m_{it}=f(x_{it},\omega_{it})$$

where function *f*(.,.) satisfies the monotonicity, so we can invert the intermediate input demand function and write

(4) $$\omega_{it}=f^{-1}(x_{it},m_{it})=h(x_{it},m_{it})$$

Referring to both the methods of OP and LP, in order to estimate the parameters, it assumes

(5) $$E(\varepsilon_{it}|\omega_{it-1},w_{it},x_{it},m_{it},w_{it-1},x_{it-1},m_{it-1},\ldots ,w_{i1},x_{i1},m_{i1})=0$$

without imposing any functional form on the control function $\omega_{it}=h(.,.)$.

Then, exploiting the Markovian nature of productivity and other assumptions which are used in LP, it supposes

(6) $$E(\omega_{it}|x_{it},w_{it-1},x_{it-1},m_{it-1},\ldots ,w_{i1},x_{i1},m_{i1})=E(\omega_{it}|\omega_{it-1})=f\left\{h(x_{it-1},m_{it-1})\right\}$$

where, as for *h*(.,.), no functional form is imposed on *f*(.,.). Therefore, two equations can be derived directly by Hypothesis (5) and (6)

(7) $$y_{it}=\alpha +w_{it}\beta +x_{it}\gamma +h(x_{it},m_{it})+v_{it}$$

(8) $$y_{it}=\alpha +w_{it}\beta +x_{it}\gamma +f\{h(x_{it-1},m_{it-1})\}+\eta_{it}$$

where $\eta_{it}=\varepsilon_{it}+\upsilon_{it}$.

In the estimation, we deal with the unknown functional form using the approach of nth-degree polynomials in $x_{it}$ and $m_{it}$. In particularly, if we assume that

(9) $$h(x_{it},m_{it})=\lambda_{0}+k(x_{it},m_{it})\lambda$$

where, k(.,.) is a 1×Q collection of functions,

(10) $$f(h)=\delta_{0}+\delta_{1}h+\delta_{2}h^{2}+\ldots +\delta_{G}h^{G}$$

Then, we get

(11) $$f(\omega_{it})=\delta_{0}+\delta_{1}\{k(x_{it-1},m_{it-1})\lambda_{1}\}+\delta_{2}{\left\{k(x_{it-1},m_{it-1})\lambda_{1}\right\}}^{2}+\ldots +\delta_{G}{\left\{k(x_{it-1},m_{it-1})\lambda_{1}\right\}}^{G}$$

To simplify, thinking about G=1 and $\delta_{1}=1$, a simple substitution in (7) and (8) yields

(12) $$y_{it}=\varsigma +w_{it}\beta +x_{it}\gamma +k(x_{it},m_{it})\lambda_{1}+v_{it}$$

(13) $$y_{it}=\theta +w_{it}\beta +x_{it}\gamma +k(x_{it-1},m_{it-1})\lambda_{1}+\eta_{it}$$

where *ζ* and *θ* are the new constant parameters. 

Then, we use the method of dynamic panel tool variables proposed by Blundell and Bond [48]. A 2(T−1) residual function vector is defined for each t>1 as

(14) $$r_{i}(\theta )=\left[\begin{array}{l} y_{i2}-\varsigma -w_{i2}\beta -x_{i2}\gamma -k(x_{i2},m_{i2})\lambda_{1}\\ y_{i2}-\theta -w_{i2}\beta -x_{i2}\gamma -k(x_{i1},m_{i1})\lambda_{1}\\ \ldots \\ \ldots \\ y_{iT}-\varsigma -w_{iT}\beta -x_{iT}\gamma -k(x_{iT},m_{iT})\lambda_{1}\\ y_{iT}-\theta -w_{iT}\beta -x_{iT}\gamma -k(x_{iT-1},m_{iT-1})\lambda_{1} \end{array}\right]$$

For each panel i, we define t−b, the last available lag (that is, when b=1 at t=2; when b=T−1 at t=T. Then, let $Z_{i}$ represent the dynamic panel instrument matrixes for each panel i:

(15) $$Z_{i}=\left[\begin{array}{llllllll} z_{i2}^{\prime } & z_{i3}^{\prime } & \cdots & z_{iT}^{\prime } & 0 & 0 & 0 & 0\\ 0 & 0 & \cdots & 0 & {\tilde{z}}_{i3}^{\prime } & 0 & 0 & 0\\ 0 & 0 & \cdots & 0 & 0 & {\tilde{z}}_{i4}^{\prime } & \cdots & 0\\ \vdots & \vdots & \cdots & \vdots & \vdots & & \ddots & 0\\ 0 & 0 & 0 & 0 & 0 & 0 & 0 & {\tilde{z}}_{iT}^{\prime }\\ 0 & 0 & 0 & 0 & 1 & 1 & 1 & 1 \end{array}\right]$$

where, the component ${\tilde{z}}_{it}^{\prime }$ is a 1×b vector, which is consisted of $z_{it-1},\ldots ,z_{it-b}$. $z_{it}=\left[\begin{array}{l} 1,x_{it},w_{it},k(x_{it},m_{it})\\ 1,x_{it},w_{it},k(x_{it-1},m_{it-1}) \end{array}\right]$.

As usual, the conditions of GMM are defined as

(16) $$E\{Z_{i}r_{i}(\theta )\}=0$$

**Figure 1:**
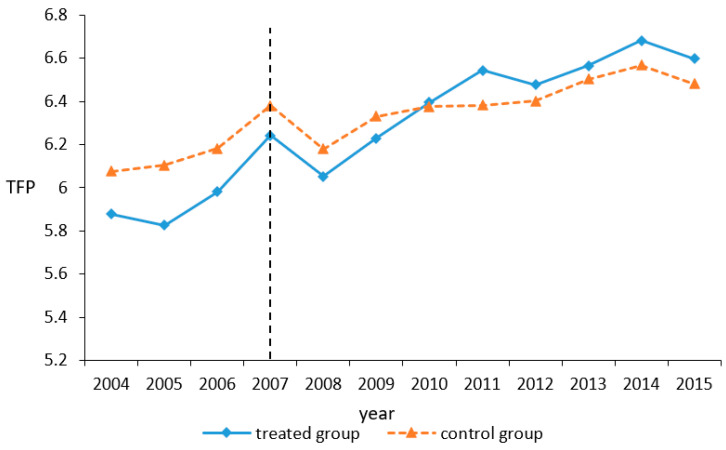
Trend of corporate total factor productivity (TFP).

Finally, based on the regression coefficient from GMM estimation, the residuals are used to measure the enterprise TFP. Figure 1 compares the trend of enterprise TFP before and after the implementation of SO_2_ ETP policy between pilot and non-pilot areas. As Figure 1 shows, the average TFP level in pilot areas is lower than that in non-pilot areas before 2007, but it has gradually surpassed that since 2010. The preliminary diagnosis shows that the SO_2_ ETP seems to promote the enterprise TFP, which provides the conditions for the quasi-natural experimental research.

### 3.4. Other Variables

For the control variables, this paper mainly considers the internal characteristics of the enterprise. (1) Enterprise size (lnta). The size of an enterprise is expressed by the logarithm of its total assets (donated in million Yuan). Schumpeter hypothesis holds that smaller firms have less innovation motivation and advantages in technological innovation, while the larger size is more beneficial to technological innovation and productivity [68]. (2) Capital labor density (cd). The capital labor intensity is equal to net value of fixed assets (donated in million Yuan) divided by the number of employees in the enterprise (donated in thousand). The flow of production factors such as capital, labor, as well as the change of the share of each factor in the same industry are of great significance to the improvement of enterprise productivity. Generally speaking, enterprises with higher capital-labor ratio pay more attention to the equipment renewal and R&D investment, which leads to improve TFP [69]. (3) Ratio of fixed assets (fr). It refers to the proportion of net fixed assets in total assets. The lower this value is, the better liquidity of enterprise assets will be. Thus, the firms carry on innovations more actively, thereby increasing TFP. (4) Nature of property right (owner). According to Yu [70], a dummy variable is used to represent the property rights of enterprise, for instance, owner is equal to 1 that means state-owned enterprises, and owner is equal to 0 that means non-state-owned enterprises. The productivity of the state-owned enterprises is relatively low because of the special Chinese system. While, the non-state-owned enterprises which formed under the market mechanism have better technical characteristics and higher productivity level. (5) Time of company foundation (age). In this paper, we use this formula of the current year minus the establishment year minus 1 to get the year of the age. (6) Capital return (roa). This indicator is expressed as the rate of assets return. It reflects the return on assets and the financial condition of enterprise [71]. Generally speaking, the better business’s financial condition, the more attention it pays to productivity improvement. (7) Capital structure (dar). The higher the debt ratio, the greater the financial risk the enterprise faces, which may restrain the productivity of the enterprise. Besides’ that, for the external factors, it mainly considers of environmental law enforcement (lnele), and uses the measurement of logarithm from the number of every provincial environmental administrative punishment cases. The productivity effect of environmental regulation is higher when the enforcement of environmental law gets greater [8].

In addition, there are two moderating variables. The first one is financing constraints (fc). There are many means of financing constraints measurement, such as the ZFC index by Cleary [72], KZ index by Lamont et Al. [73], and WW index [74]. However, these methods, which rely on endogenous financial indicators, but are not related to financing constraints directly, make their research conclusions biased. To avoid this deficiency, Hadlock and Pierce [75] redesigned the financing constraint variable (SA index). The SA index only uses two variables of firm size and age that do not change much but have strong exogenous character to construct the model. The specific formula is $SA\;index=-0.737\times size+0.043\times size^{2}-0.040\times age$. Where, size is the natural logarithm of the total assets; age is the enterprise’s established time. The larger SA index means the more serious of financing constraint. The second one is the bargaining power (bar). The bargaining power of enterprises in the implementation of environmental regulations comes from the degree of their contribution on the local economy and officials’ performance evaluation. Based on Li and Chen’s practice [44], this paper selects total output (donated in hundred Yuan) and takes its logarithm to measure the bargaining power of the enterprises. In addition, we also calculate TFP that expressed as TFP(LP) by LP semi-parametric method, in order to verify the robustness of the empirical results. Besides, we replace the lower and upper outliers of some variables with 1% and 99% quantile respectively. The descriptive statistics of variables can be found in Table 1.

## 4. Results

### 4.1. Result of PSM 

**Figure A1:**
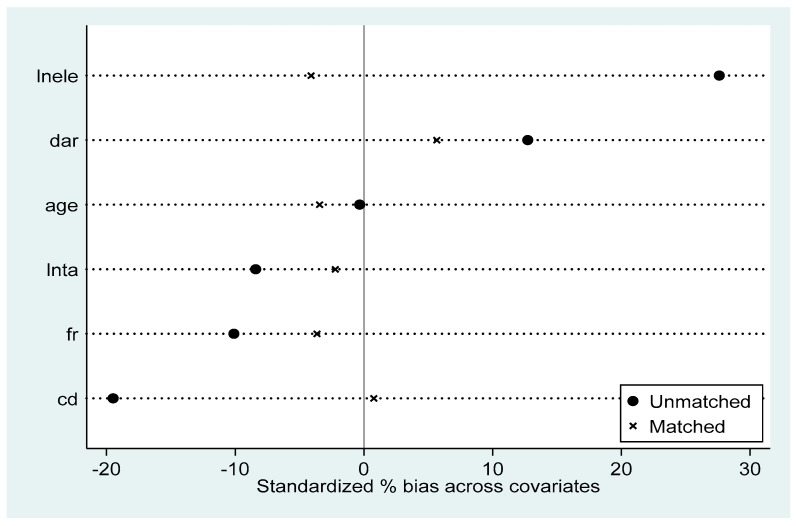
Deviation of covariates before and after matching.

Before using DID method, all the control variables (except for roa) mentioned above are chosen as covariables to match the samples. In order to guarantee the validity of the PSM-DID method, a series of corresponding tests are carried out on the matching grouping results. First, we are going to check out whether the distribution of the variables in treated and control groups becomes balanced after matching. Generally, if there is no significant difference between the mean of covariates before and after matching, but there is significant difference in *t* test (significant before and not after matching), then the application of PSM-DID method is supported. The covariate test results (see Table A1 in Appendix A) show that there is no significant difference in the mean of covariates, and all the covariates in the *t* test become nonsignificant after the PSM. This indicates the PSM-DID method is appropriate. Second step is checking the deviation of covariates before and after matching (see Figure A1 in Appendix A). It can be seen that the after-matched values are close to zero and within 10% of the standard deviation, which means the matching result is good by nearest-neighbor matching method. The final matching result leaves 3099 samples, by deleting 21 samples that did not match.

### 4.2. Baseline Estimations

The baseline regression result of SO_2_ ETP on enterprise TFP for full sample is shown in Table 2 with four columns. The Column (1) controls the fixed effects of industry, area, and year. The Column (2) adds control variables based on Column (1). The results show that the coefficients of time*treated are 0.235 and 0.223 respectively (significant under 1% significance level), which indicates that SO_2_ ETP improves enterprise TFP significantly for the full sample. Based on the Columns (1) and (2), the Columns (3) and (4) consider individual characteristics that do not change over time, respectively. Both of regression coefficients of time*treated in Columns (3) and (4) are 0.234 (significant at 1% significance level). In addition, the value of the coefficient reflects the net effect of emission trading policy implementation, which mean that the implementation of ETP improves the overall TFP by 23.4%. Above that, it tells that the regression results with individual fixed effect or without are basically consistent. Therefore, SO_2_ ETP improves the overall TFP significantly, which proves the Hypothesis 1. Besides, the results of each model are consistent, and the coefficients of control variables are significant, and the symbol of coefficients of control variables are reasonable, which means that the model has certain robustness.

The results suggest that SO_2_ ETP, the largest market-type environmental regulation supports the strong Porter hypothesis on the whole. From the theory, in the short time, ETP may bring some cost pressure. However, in the long run, it provides enterprises with a variety of emission reduction options, compliance pressure, and economic compensation effects, thus stimulate enterprises to improve the technology of pollution control and production. 

### 4.3. Robustness Tests

In order to get more reliable results, in this subsection, we carry out a series of robustness tests. First, the parallel trend test is applied to the group of treated and control. Second, we re-estimate the benchmark DID model with the TFP measured by the LP semi-parametric method. Third, the placebo test is performed by constructing the virtual treated and control group randomly. Finally, a set of experiments is reconstructed to test by the DDD method.

#### 4.3.1. Parallel Trend Test

The consistency of DID estimates is based on the assumption that the treated and control group satisfy the parallel trend hypothesis. That is to say, before the policy intervention, the change trend of the outcome variables in the two group is consistent. Figure 1 has shown that the average TFP of both groups has roughly the same trend before the policy implementation (before 2007). But, in order to make the DID results more rigorous, referring to the practice of Alder et al. and Beck et al. [76,77], we construct the following model:

(17) $$TFP_{it}=\alpha_{0}+\sum_{t=2004}^{2015}\beta_{t}treated*year^{t}+X_{it}\lambda +\mu_{i}+\theta_{j}+\upsilon_{k}+\gamma_{t}+\varepsilon_{ijkt,}$$

where 2007 is the base year, $year^{t}$ is the time dummy variable, $\beta_{t}$ represents a series of estimated value during 2004–2015, the other variable definition is consistent with the benchmark regression model (1). If the regression coefficients $\beta_{2004}-\beta_{2006}$ are not significant, then the treated and control groups satisfy the parallel trend test.

**Figure 2:**
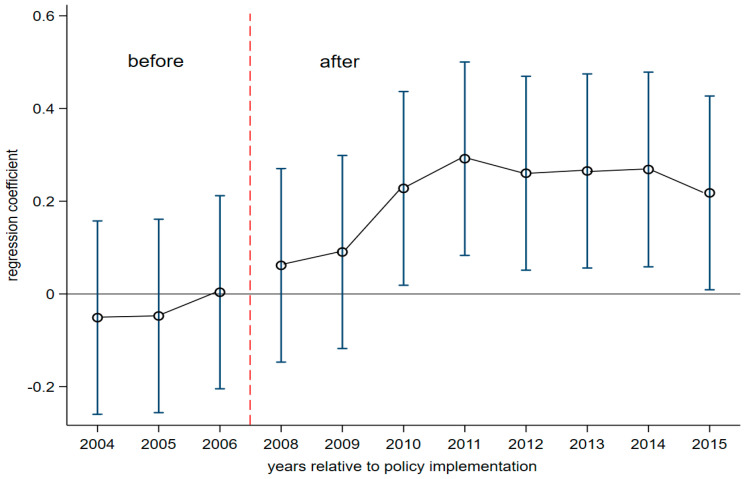
Dynamic effect of differences-in-differences (DID) model. Notes: The parameter estimates (dots) and corresponding 95% confidence intervals (line) based on model (17).

The estimation of the dynamic effects of SO_2_ ETP on enterprise TFP is shown in Table 3. The fixed effects of industry, area, time and individual are controlled by Column (1). Column (2) adds a control variable based on Column (1). The results showed that the dynamic effect regression Coefficients $\beta_{t}$ from 2004 to 2006 are not significant. It indicates that there is no significant difference between treated and control group before the implementation of SO_2_ ETP, i.e., it satisfies the parallel trend assumption. In order to get better illustrating of the result of parallel trend test, Figure 2 plots the point estimates of the coefficients $\beta_{t}$ and the corresponding 95% confidence interval estimates. It also shows that the treated and control groups meet the requirements of parallel trend assumption. In addition, from the fourth year after the implementation of this environmental policy, the parameters of $treated*year^{t}$ become significant. It reflects that the effect of policy implementation on TFP has a certain lag.

#### 4.3.2. Alternative Variable Test

In order to test the robustness of benchmark regression results, we adopt LP semi-parametric method to measure TFP and estimate the effect of SO_2_ ETP on TFP. The result is shown in Table 4 with four columns. In Columns (1), (2), (3), and (4), the regression coefficient of time*treated are significantly positive. In addition, comparing with Table 2, we can find that the regression estimations under two measurement method of TFP are basically the same. These show changing the measurement of dependent variable will not change the estimation result.

#### 4.3.3. Placebo Test

**Figure 3:**
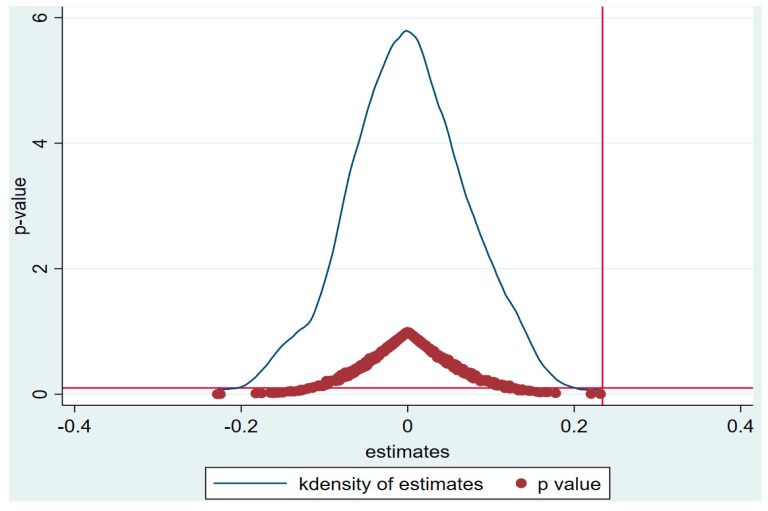
Result of placebo test.

In order to further exclude the differences of TFP of enterprises between the pilot and non-pilot because of the various other factors, the placebo test is conducted by assigning the treated and control group randomly [78]. This method ensures the constructed independent variable of time*treated have no effect on enterprise TFP. In the sample of baseline model, 260 enterprises are involved, including 101 in pilot provinces (treated group) and 159 in non-pilot provinces (control group). Based on these 260 enterprises, this paper generates corresponding number of samples to make up treated and control group randomly. Then, based on the new samples, the benchmark DID model (1) is estimated. Then, they are repeated 500 times. We plot the kernel densities of 500 estimated coefficients and their *p* values, as shown in Figure 3. On the one hand, the estimated coefficients are all concentrated near the zero point, which shows that the regression results are robust. On the other hand, the vertical line, which displays the real net effect of policy implementation in the benchmark regression model (1), is on the right side of the 500 estimates values. It also indicates that the benchmark regression result is robust.

#### 4.3.4. DDD Test

In view of the complexity of real-world social systems, other environmental policies in these periods may have impact on the assessment of policy effectiveness. That is to say, the results of the DID model may be biased. Actually, there are some contemporaneous environmental policies, such as the pilot policy of carbon emission rights trading in 2011 in seven provinces, PM2.5 monitoring policy in 2012, special emission allowances policy for air pollutants in 2013, and the pilot policy on water rights trading in 2014 in seven provinces. In order to eliminate the errors caused by the above policies, the DDD method is used to verify the results. In this paper, the manufacturing enterprises which belong to non-SO_2_ emission industries are selected as the other treated and control groups of the DDD model. Based on the benchmark regression Equation (1), the DDD model is constructed as follows:

(18) $$\begin{array}{ll} TFP_{it} & =\alpha_{0}+\alpha_{1}time_{t}*treated_{i}*polluting_{i}+\alpha_{2}time_{t}*treated_{i}+\alpha_{3}time_{t}*polluting_{i}\\ & +\alpha_{4}treated_{i}*polluting_{i}+X_{it}\lambda +\mu_{i}+\theta_{j}+\upsilon_{k}+\gamma_{t}+\varepsilon_{ijkt,} \end{array}$$

where, polluting is a dummy variable. If the sample enterprise belongs to SO_2_ emission enterprise, it is equal to 1; otherwise, it is equal to 0. $\alpha_{1}$ represents the net effect of the implementation of ETP system. Meanwhile, the two interaction terms like time*treated, time*polluting and treated*polluting are also added, in order to guarantee the real economic meaning of $\alpha_{1}$. In the robustness test, we are concerned about the coefficient $\alpha_{1}$. If $\alpha_{1}$ is significant, it means that SO_2_ ETP promotes the overall TFP. In addition, the definition of other variables is in line with Equation (1).

Table 5 shows the estimates of DDD model. In Columns (2), (3), and (4), all the regression coefficients $\alpha_{1}$ of SO_2_ ETP are significantly positive, which indicates that SO_2_ ETP improves the overall TFP significantly.

## 5. Heterogeneity and Moderating Effects Analysis

### 5.1. Heterogeneity Analysis

The existing literature mostly studies the heterogeneous effect of environmental regulation on enterprise TFP from the aspects of property nature and scale, and the conclusion is consistent. However, only a few literatures have found that environmental regulation has heterogeneous impact on enterprise TFP under different TFP levels [5,15]. Moreover, they reached different conclusions because of the different types of environmental regulations, research methods, and samples. Peng et al. [5] show that ETP has positive contribution to enterprise TFP at different quantile of TFP, and enterprise TFP with higher level of TFP gets stronger effect. In contrast, Albrizio et al. [15] concluded that the stricter environmental regulation increases the productivity for enterprises with high TFP and decreases the productivity for enterprises with low TFP. Actually, the estimation results may be affected by the extreme value because distribution of enterprise TFP may have a large degree of dispersion, and there are many structural changes in the real economy. Therefore, based on the benchmark regression Equation (1), following the way of Li et al. [79] and Broni et al. [80], the heterogeneous effects of SO_2_ ETP on enterprise TFP at different quantiles of TFP are gained by unconditional panel quantile fixed effect regression model.

Estimates at 10–90% quantile level of enterprise TFP is showed in Table 6. It can be seen that SO_2_ ETP has no significant influence on enterprise TFP at 10%–30% quantile of TFP. Then, the effect is significant at 40%–80% quantile of TFP, and increases at first and then decreases. Moreover, the influence of SO_2_ ETP on enterprise TFP become insignificant when at 90% quantile of TFP. This just supports Hypothesis 2.

### 5.2. Moderating Effects Analysis

The results of baseline regression in Section 4.1 have proved that SO_2_ ETP has a positive effect on the overall TFP. But this effect is also restricted by other factors. In order to better realize the productivity effect of ETP, this part examines the moderating mechanism from financing constraints and bargaining power.

#### 5.2.1. Moderating Effect of Financing Constraint

Financing is an important factor to improve business and environmental behavior, so financing constraints may have an impact on the relationship between SO_2_ ETP and enterprise TFP. In order to test the moderating effect of financing constraints, this paper constructs the following model:

(19) $$\begin{array}{ll} TFP_{it} & =\beta_{0}+\beta_{1}time_{t}*treated_{i}+\beta_{2}fc_{it}+\beta_{3}time_{t}*treated_{i}*fc_{it}+X_{it}\lambda +\mu_{i}+\theta_{j}+\upsilon_{k}\\ & +\gamma_{t}+\varepsilon_{ijkt,} \end{array}$$

where fc represents financing constraints, which is measured by SA index; other variables are defined in accordance with Equation (1). The model includes not only ETP policy implementation, financing constraint, but also their interaction. If the regression coefficient $\beta_{3}$ is significant, then financing constraint has a moderating effect. Meanwhile, if negative, it indicates that the financing constraint has the reverse adjustment effect.

Columns (1) and (2) in Table 7 show the moderating effect of financing constraints on the impact of SO_2_ ETP on enterprise TFP for the full sample. The results showed the regression coefficients of the interaction term of time*treated*fc in Columns (1) and (2) are −0.468, −0.358 (significant under 1% significance level) respectively, and the coefficient of the main effect of time*treated is significantly positive. This indicates that the financing constraint has a significant negative effect on the relationship between SO_2_ ETP and enterprise TFP. That is, if the degree of financing constraint gets higher, the effect of SO_2_ ETP on enterprise TFP becomes weaker. This proves Hypothesis 3 is right.

In addition, the heterogeneous moderating effects of financing constraints on the impact of SO_2_ ETP on TFP is examined from the perspective of property right nature. Columns (3), (4), (5), and (6) in Table 7 show the test results of the moderating effect of financing constraints for the state-owned and non-state-owned enterprises respectively. Among them, the regression coefficients $\beta_{3}$ of time*treated*fc in Columns (3) and (4) are insignificant, but significant in Columns (5) and (6). It shows that the financing constraints have a significant moderating effect on the impact of SO_2_ ETP on TFP for non-state-owned enterprise, while not significant for state-owned enterprises. This is related to the inconsistency of financing constraints degree between state-owned and non-state-owned enterprises. State-owned enterprises can obtain additional funds such as government subsidies, and also more external funds through government credit intervention. Thus, they have a low degree of financing constraints. While, non-state-owned enterprises have more obstacles and difficulties in financing. Therefore, the moderating effects of financing constraints are heterogeneous between state-owned and non-state-owned enterprises.

#### 5.2.2. Moderating Effect of Bargaining Power

When the heavily polluting enterprises contribute a lot to the local economic growth, the local government offers them preferential treatment on environmental cost. Under this circumstance, these companies use bargaining chip for their importance on local government and obtain special treatment even in the face of strict environmental regulations. Thus, it lowers the economic effect of environmental regulations. Based on Equation (1), this paper constructs the following model to test the moderating effect of bargaining power:

(20) $$\begin{array}{ll} TFP_{it} & =\beta_{0}+\beta_{1}time_{t}*treated_{i}+\beta_{2}bar_{it}+\beta_{3}time_{t}*treated_{i}\times bar_{it}+X_{it}\lambda +\mu_{i}+\theta_{j}+\upsilon_{k}\\ & +\gamma_{t}+\varepsilon_{ijkt,} \end{array}$$

where bar represents the bargaining power; other variables are defined in accordance with Equation (1). In the implementation of environmental regulation, the enterprises’ bargaining power to government comes from their contributions to the local economy and officials’ performance evaluation. Li and Chen [2] selected three variables of industrial output value, total tax payment, and employees number to measure the bargaining power of enterprises respectively, and found that all these variables have significant moderating effects. Therefore, we use the logarithm of the total output to measure the bargaining power of enterprises. If the regression coefficient $\beta_{3}$ of the interaction term of time*treated*bar is significant, it shows the bargaining power of enterprise has a moderating effect. Meanwhile, if negative, it indicates the reverse moderating effect. 

In Table 8, Columns (1) and (2) show the results of the moderating effects for the full sample. It can be seen that the regression coefficients of the interaction term of time*treated*bar in Columns (1) and (2) are −0.066, −0.063 (significant under 5% and 1% significance level respectively). Furthermore, its main effect of time*treated is significantly positive. Thus, we confirm that the bargaining power of enterprises has a significant negative moderating effect on the relationship between SO_2_ ETP and enterprise TFP. This has supported the Hypothesis 4.

Moreover, the heterogeneous moderating effects of bargaining power on the impact of SO_2_ ETP on TFP is examined from the perspective of property right nature. Columns (3), (4), (5), and (6) in Table 8 show the test results of the moderating effect of bargaining power for the state-owned and non-state-owned enterprises respectively. Among them, the regression coefficients $\beta_{3}$ of time*treated*bar in Columns (3) and (4) are significant, but insignificant in Columns (5) and (6). It indicates that the moderating effect of bargaining power is significant for state-owned enterprises, while insignificant for non- state-owned enterprises. The heterogeneity is directly related to current political system environment in China. On the one hand, as the investor and supporter of state-owned enterprises, government can exert a significant influence on their personnel appointment and removal and business decisions. On the other hand, state-owned enterprises determine local economic growth and tax revenue to some extent, and their profits even support local public supplies. Therefore, the state-owned enterprises obtain a large degree of exemption from environmental regulation, which makes the moderating effect of their bargaining power significant. While, the non-state-owned enterprises do not have these unique advantages.

## 6. Conclusions

Whether environmental regulation can realize the win-win of economic benefit and environmental performance has become the topic of common concern of the academics and the government. Is there a contradiction between strengthening environmental regulation and improving the quality of economic development in the context of severe environmental pollution and increasing resource constraints? The answer is no. Based on the data of SO_2_ emission enterprises in Chinese listed manufacturing industry, this paper constructs an identification framework of PSM-DID to study the impact of SO_2_ ETP on enterprises TFP. The main conclusions are as follows:

First, SO_2_ ETP supports the strong Porter hypothesis on the whole. On the one hand, estimates from the benchmark regression model show that SO_2_ ETP can significantly increase the enterprises TFP for the full sample. On the other hand, a series of robustness tests, such as parallel trend test, revaluation of TFP using LP method, placebo test, and DDD model, all show that SO_2_ ETP has a significant productivity effect for the full sample.

Second, the influence of SO_2_ ETP on TFP is heterogeneous at different quantile level of TFP. From the results of the unconditional quantile panel fixed effect regression, the influence of SO_2_ ETP on enterprises is significant at 40%–80% quantiles of TFP, and increases first and then decreases. However, it is not significant at 10%–30% quantiles level and higher than 90% quantiles.

In addition, the financing constraints and bargaining power have significant negative moderating effects on the relationship between SO_2_ ETP and TFP, and the moderating effects are heterogeneous under different property right nature. To be specific, the moderating effect of financing constraints is significant for non-state-owned company but insignificant for state-owned company. While, the moderating effect of bargaining power is significant for state-owned enterprises, but insignificant for non-state-owned enterprises.

Accordingly, the following policy implications can be pursued to improve the economic effect of ETP as follows: first, the optimization of market-based environmental regulation should be further implemented. Specially, the first one is to locate the pollutant emitters accurately and activate the transaction and price discovery mechanism of the emission permit market [81]. Second, the government should supplement policy tools of price, tax, and subsidy in the implementation of ETP, and thus provide flexible institutional security and economic incentives for the green innovation of enterprises with different levels of TFP.

Then, the financing mechanism on environmental protection should be established gradually. On the one hand, the government should encourage the external financial institutions like bank to enter into long-term cooperative relations with enterprises that actively fulfill their environmental protection responsibilities [82]. On the other hand, the government should broaden the financing channels for enterprises, especially non-state-owned enterprises, through building green technology financial system vigorously, developing and making use of new financing tools such as green credit and green bonds.

Additionally, the bargaining power of enterprises on environment should be weakened. First, the implementation of emissions trading and other environmental performance-related issues should be included in the performance of government officials. Second, intervention of local governments in the implementation of environmental policies should be weakened. Besides, the supervision and implement administrative punishment for enterprises, especially for non-state-owned enterprises should be strengthened. 

## Figures and Tables

**Table 1:** Descriptive statistics.

Variable	Obs	Mean	Std. Dev.	Min	Max
TFP	3120	6.327	0.922	3.046	8.539
TFP(LP)	3120	4.850	0.875	1.680	7.072
cd	3120	0.927	1.485	0.025	8.294
fr	3120	0.381	0.187	0.023	0.782
lnta	3120	8.386	1.292	5.198	11.451
age	3120	15.642	6.409	3	73
owner	3120	0.596	0.491	0	1
roa	3120	0.025	0.064	−0.279	0.197
dar	3120	0.555	0.208	0.107	1.424
lnele	3120	7.866	1.192	4.094	10.068
fc	3120	3.710	0.261	2.949	5.721
bar	3120	17.052	1.454	11.721	21.409

**Table 2:** Baseline estimations effect of SO_2_ emissions trading polit (ETP) on TFP.

TFP	(1)	(2)	(3)	(4)
time*treated	0.235 ***	0.223 ***	0.234 ***	0.234 ***
	(3.73)	(4.51)	(4.64)	(5.05)
cd		0.047 ***		0.042 **
		(3.94)		(2.41)
lnta		0.340 ***		0.179 ***
		(26.69)		(7.61)
fr		−0.989 ***		−0.910 ***
		(−11.65)		(−7.70)
age		−0.001		0.023 ***
		(−0.23)		(3.98)
owner		0.029		
		(1.03)		
roa		4.671 ***		3.679 ***
		(21.45)		(16.34)
dar		−0.378 ***		−0.322 ***
		(−5.21)		(−3.37)
lnele		0.063 ***		0.069 ***
		(2.86)		(3.34)
_cons	6.306 ***	3.420 ***	6.016 ***	4.223 ***
	(32.97)	(14.72)	(149.81)	(17.94)
industry fe	yes	yes	yes	yes
area fe	yes	yes	yes	yes
year fe	yes	yes	yes	yes
individual fe	no	no	yes	yes
observations	3099	3099	3099	3099
R-squared	0.243	0.544	0.039	0.423

Notes: fe stands for fixed effect; *, **, *** stands for the significance level of 10%, 5%, 1% respectively. T values are reported in the parenthesis.

**Table 3:** Dynamic effect of SO_2_ emissions trading pilot (ETP) on TFP.

TFP	(1)	(2)
treated * year ^2004^	−0.072 (−0.62)	−0.051 (−0.48)
treated * year ^2005^	−0.098 (−0.84)	−0.047 (−0.45)
treated * year ^2006^	−0.037 (−0.32)	0.004 (0.04)
treated * year ^2007^	omitted	omitted
treated * year ^2008^	0.035 (0.30)	0.062 (0.58)
treated * year ^2009^	0.048 (0.41)	0.090 (0.85)
treated * year ^2010^	0.166 (1.43)	0.228 ** (2.14)
treated * year ^2011^	0.273 ** (2.34)	0.292 *** (2.74)
treated * year ^2012^	0.226 * (1.94)	0.260 ** (2.44)
treated * year ^2013^	0.216 * (1.86)	0.265 ** (2.49)
treated * year ^2014^	0258 ** (2.22)	0.269 ** (2.51)
treated * year ^2015^	0.236 ** (2.03)	0.218 ** (2.05)
Constant	6.045 *** (99.58)	4.258 (17.24)
Control variables	no	added
industry fe	yes	yes
area fe	yes	yes
year fe	yes	yes
individual fe	yes	yes
observations	3099	3099
R-squared	0.100	0.253

Notes: fe stands for fixed effect; *, **, *** stands for the significance level of 10%, 5%, 1% respectively. T values are reported in the parenthesis.

**Table 4:** Baseline estimation effect of SO_2_ emissions trading pilot (ETP) on TFP (LP).

TFP	(1)	(2)	(3)	(4)
time*treated	0.244 ***	0.235 ***	0.243 ***	0.245 ***
	(4.02)	(4.75)	(4.83)	(5.29)
cd		0.092 ***		0.082 ***
		(7.66)		(4.74)
lnta		0.217 ***		0.070 ***
		(17.02)		(2.96)
fr		−1.258 ***		−1.161 ***
		(−14.79)		(−9.82)
age		−0.001		0.024 ***
		(−0.63)		(4.14)
owner		0.025		omitted
		(0.87)		
roa		4.665 ***		3.662 ***
		(21.39)		(16.26)
dar		−0.389 ***		−0.326 ***
		(−5.34)		(−3.41)
lnele		0.053 **		0.059 ***
		(2.39)		(2.86)
_cons	4.887 ***	3.152 ***	4.598 ***	3.785 ***
	(26.57)	(13.54)	(114.65)	(16.07)
industry fe	yes	yes	yes	yes
area fe	yes	yes	yes	yes
year fe	yes	yes	yes	yes
individual fe	no	no	yes	yes
observations	3099	3099	3099	3099
R-squared	0.223	0.492	0.028	0.352

Notes: fe stands for fixed effect; *, **, *** stands for the significance level of 10%, 5%, 1% respectively. T values are reported in the parenthesis.

**Table 5:** Estimation results of differences-in-differences-in-differences (DDD) model.

TFP	(1)	(2)	(3)	(4)
time*treated*polluting	0.078	0.194 ***	0.389 ***	0.358 ***
	(0.90)	(2.97)	(5.36)	(5.45)
time*treated	−0.001	−0.060	−0.155 ***	−0.137 ***
	(−0.02)	(−1.23)	(−3.00)	(−2.93)
time*polluting	−0.097 **	−0.147 ***	−0.408 ***	−0.316 ***
	(−2.17)	(−4.30)	(−9.06)	(−7.70)
treated*polluting	0.097	−0.092 *	0.000	0.000
	(1.43)	(−1.79)	(.)	(.)
cd		0.044 ***		0.032 **
		(4.08)		(2.13)
lnta		0.360 ***		0.253 ***
		(41.97)		(15.37)
fr		−1.270 ***		−1.186 ***
		(−19.54)		(−12.89)
age		−0.001		0.043 ***
		(−0.29)		(9.63)
owner		0.018		0.000
		(0.94)		(.)
roa		4.597 ***		3.444 ***
		(31.40)		(22.78)
dar		−0.156 ***		−0.198 ***
		(−3.34)		(−3.06)
lnele		0.035 **		0.037 **
		(2.16)		(2.49)
_cons	5.860 ***	3.461 ***	5.962 ***	3.731 ***
	(34.04)	(19.25)	(206.96)	(22.64)
industry fe	yes	yes	yes	yes
area fe	yes	yes	yes	yes
year fe	yes	yes	yes	yes
individual fe	no	yes	no	yes
observations	6183	6183	6183	6183
R-squared	0.187	0.538	0.072	0.425

Notes: fe stands for fixed effect; *, **, *** stands for the significance level of 10%, 5%, 1% respectively. T values are reported in the parenthesis.

**Table 6:** Estimation results under different quantile level of enterprise TFP.

TFP	(1)	(2)	(3)	(4)	(5)	(6)	(7)	(8)	(9)
	10th	20th	30th	40th	50th	60th	70th	80th	90th
time*treated	0.190	0.125	0.142	0.238 **	0.254 ***	0.256 ***	0.264 ***	0.195 **	0.095
	(0.88)	(0.96)	(1.36)	(2.44)	(2.77)	(3.04)	(2.91)	(1.99)	(0.77)
cd	0.018	0.012	0.046	0.013	0.015	0.033	0.052	0.073	0.095
	(0.33)	(0.34)	(1.29)	(0.39)	(0.49)	(0.86)	(1.50)	(1.58)	(1.43)
lnta	0.274 *	0.173 **	0.137 **	0.158 ***	0.176 ***	0.181 ***	0.095 **	0.090 *	0.031
	(1.93)	(2.36)	(2.33)	(2.91)	(3.50)	(3.78)	(1.98)	(1.87)	(0.45)
fr	−1.290 **	−1.030 ***	−1.093 ***	−1.005 ***	−0.891 ***	−0.914 ***	−0.804 ***	−0.809 ***	−0.832 ***
	(−2.11)	(−3.15)	(−4.19)	(−4.00)	(−4.11)	(−4.14)	(−3.57)	(−3.34)	(−2.91)
age	−0.011	0.031 **	0.046 ***	0.046 ***	0.039 ***	0.028 ***	0.040 ***	0.037 ***	0.048 ***
	(−0.45)	(2.13)	(4.23)	(4.63)	(4.21)	(3.21)	(4.55)	(3.77)	(3.62)
owner	0.000	0.000	0.000	0.000	0.000	0.000	0.000	0.000	0.000
	(.)	(.)	(.)	(.)	(.)	(.)	(.)	(.)	(.)
roa	5.171 ***	4.324 ***	3.258 ***	3.500 ***	3.356 ***	3.136 ***	3.277 ***	2.824 ***	3.103 ***
	(3.64)	(5.31)	(5.24)	(5.88)	(6.30)	(6.46)	(6.75)	(5.70)	(4.38)
dar	−1.131 **	−0.535 *	−0.409 **	−0.248	−0.192	−0.115	−0.140	−0.229	0.022
	(−2.50)	(−1.91)	(−2.02)	(−1.21)	(−1.03)	(−0.68)	(−0.76)	(−1.25)	(0.10)
lnele	0.086	0.006	0.016	0.039	0.052	0.034	0.042	0.047	0.091
	(1.21)	(0.15)	(0.43)	(1.13)	(1.48)	(1.06)	(1.22)	(1.26)	(1.46)
_cons	3.407 ***	4.084 ***	4.304 ***	4.118 ***	4.150 ***	4.585 ***	5.244 ***	5.609 ***	5.850 ***
	(3.15)	(7.01)	(9.18)	(8.88)	(9.34)	(11.38)	(12.38)	(11.85)	(8.07)
industry fe	yes	yes	yes	yes	yes	yes	yes	yes	yes
area fe	yes	yes	yes	yes	yes	yes	yes	yes	yes
year fe	yes	yes	yes	yes	yes	yes	yes	yes	yes
individual fe	yes	yes	yes	yes	yes	yes	yes	yes	yes
observations	3099	3099	3099	3099	3099	3099	3099	3099	3099
R-squared	0.074	0.135	0.185	0.203	0.207	0.182	0.157	0.127	0.079

Notes: fe stands for fixed effect; *, **, *** stands for the significance level of 10%, 5%, 1% respectively. T values are reported in the parenthesis.

**Table 7:** Results of moderating effect of financing constraint.

TFP	(1)	(2)	(3)	(4)	(5)	(6)
	Full	Full	SOE	SOE	Non-SOE	Non-SOE
time*treated	0.162 ***	0.188 ***	0.220 ***	0.208 ***	0.064	0.133 *
	(3.17)	(3.97)	(3.36)	(3.56)	(0.78)	(1.68)
fc	−2.027 ***	−1.133 ***	−1.424 ***	−0.620 **	−2.291 ***	−1.324 ***
	(−10.24)	(−5.84)	(−4.81)	(−2.30)	(−8.45)	(−4.47)
time*treated*fc	−0.468 ***	−0.358 ***	−0.226	−0.096	−0.796 ***	−0.740 ***
	(−3.54)	(−2.91)	(−1.36)	(−0.65)	(−3.64)	(−3.50)
cd		0.048 ***		0.044 **		0.059*
		(2.81)		(2.24)		(1.81)
lnta		0.136 ***		0.094 ***		0.164 ***
		(5.60)		(2.73)		(4.48)
fr		−0.911 ***		−0.879 ***		−0.905 ***
		(−7.76)		(−6.32)		(−4.29)
age		−0.020 **		0.003		−0.021
		(−2.16)		(0.22)		(−1.51)
owner		0.000		0.000		0.000
		(.)		(.)		(.)
roa		3.532 ***		4.635 ***		2.427 ***
		(15.71)		(14.77)		(7.46)
dar		−0.252 ***		−0.464 ***		0.055
		(−2.65)		(−3.49)		(0.40)
lnele		0.075 ***		0.050 *		0.100 ***
		(3.66)		(1.87)		(3.14)
_cons	6.454 ***	5.144 ***	6.411 ***	5.471 ***	6.393 ***	4.554 ***
	(111.14)	(18.25)	(80.98)	(15.19)	(72.45)	(10.17)
industry fe	yes	yes	yes	yes	yes	yes
area fe	yes	yes	yes	yes	yes	yes
year fe	yes	yes	yes	yes	yes	yes
individual fe	yes	yes	yes	yes	yes	yes
Observations	3099	3099	1848	1848	1251	1251
R-squared	0.134	0.261	0.088	0.278	0.215	0.289

Notes: fe stands for fixed effect; *, **, *** stands for the significance level of 10%, 5%, 1% respectively. T values are reported in the parenthesis. Full donates full sample, SOE donates state-owned enterprises, non-SOE donates non-state-owned enterprises. Financing constraints (fc) is centralized in order to avoid multicollinearity.

**Table 8:** Results of moderating effect of bargaining power.

TFP	(1)	(2)	(3)	(4)	(5)	(6)
	Full	Full	SOE	SOE	Non-SOE	Non-SOE
time*treated	0.236 ***	0.239 ***	0.324 ***	0.242 ***	0.126 *	0.208 ***
	(4.86)	(5.42)	(5.00)	(4.29)	(1.72)	(2.97)
bar	0.489 ***	0.515 ***	0.481 ***	0.523 ***	0.469 ***	0.489 ***
	(21.51)	(24.68)	(14.14)	(17.21)	(15.22)	(16.55)
time*treated*bar	−0.066 **	−0.063 ***	−0.153 ***	−0.095 ***	0.040	0.007
	(−2.51)	(−2.66)	(−4.31)	(−3.08)	(1.03)	(0.20)
cd		0.033 **		0.034*		0.033
		(2.13)		(1.92)		(1.13)
fr		−1.249 ***		−1.263 ***		−1.224 ***
		(−11.52)		(−9.67)		(−6.37)
age		−0.017 ***		−0.022 ***		−0.002
		(−3.27)		(−3.27)		(−0.23)
owner		0.000		0.000		0.000
		(.)		(.)		(.)
roa		3.530 ***		4.324 ***		2.566 ***
		(17.22)		(15.03)		(8.65)
dar		−0.360 ***		−0.608 ***		−0.060
		(−4.15)		(−5.01)		(−0.48)
lnele		0.055 ***		0.047 *		0.060 **
		(2.90)		(1.92)		(2.08)
cons	6.302 ***	6.570 ***	6.316 ***	6.807 ***	6.257 ***	6.164 ***
	(160.04)	(36.66)	(126.50)	(28.90)	(98.94)	(22.33)
industry fe	yes	yes	yes	yes	yes	yes
area fe	yes	yes	yes	yes	yes	yes
year fe	yes	yes	yes	yes	yes	yes
individual fe	yes	yes	yes	yes	yes	yes
Observations	3099	3099	1848	1848	1251	1251
R-squared	0.231	0.378	0.173	0.382	0.328	0.407

Notes: fe stands for fixed effect; *, **, *** stands for the significance level of 10%, 5%, 1% respectively. T values are reported in the parenthesis. Full denotes full sample, SOE denotes state-owned enterprises, non-SOE denotes non-state-owned enterprises. Bargaining power (bar) are centralized in order to avoid multicollinearity.

## Appendix A

**Table A1:** Equilibrium test of covariates before and after matching.

Variable	Unmatched	Mean			%Reduct	*t*-Test		V(T)/V(C)
	Matched	Treated	Control	%bias	|bias|	t	*p* > |t|	
cd	U	0.76	1.03	−19.5		−5.09	0.00	0.45 *
	M	0.76	0.75	0.8	96.1	0.25	0.81	1.16 *
lnta	U	8.32	8.43	−8.4		−2.27	0.02	0.82 *
	M	8.32	8.35	−2.2	73.5	−0.56	0.58	0.87 *
fr	U	0.37	0.39	−10.1		−2.74	0.01	0.91
	M	0.37	0.38	−3.7	63.7	−0.92	0.36	0.98
age	U	15.63	15.65	−0.3		−0.09	0.93	1.88 *
	M	15.63	15.86	−3.5	−931.8	−0.85	0.40	1.83 *
dar	U	0.57	0.55	12.7		3.41	0.00	0.74 *
	M	0.57	0.56	5.6	55.6	1.41	0.16	0.78 *
lnele	U	8.06	7.74	27.6		7.21	0.00	0.44 *
	M	8.06	8.10	−4.1	85.1	−1.12	0.26	0.60 *

Note: * stands for the significance level of 10%, 5%, 1% respectively.
